# The Practice of Teaching and Scientific Research on Cadaveric Material Remains Crucial for Medical Education

**DOI:** 10.3390/clinpract13050095

**Published:** 2023-09-01

**Authors:** Giorgio Bolino, Vittorio Fineschi, Camilla Cecannecchia, Gianpiero D’Antonio, Paola Frati

**Affiliations:** Department of Anatomical, Histological, Forensic and Orthopaedic Sciences, Sapienza University of Rome, Viale Regina Elena 336, 00161 Rome, Italy; giorgio.bolino@uniroma1.it (G.B.); camilla.cecannecchia@uniroma1.it (C.C.); gianpiero.dantonio@uniroma1.it (G.D.); paola.frati@uniroma1.it (P.F.)

**Keywords:** autopsy, body donation, surgical training, scientific research, law dissemination, anatomy teaching

## Abstract

The practice of teaching and scientific research on cadaveric material remains crucial for medical education, especially in surgical disciplines. However, in Italy, this practice has been neglected due to legislative insufficiency and financial constraints. Although innovative methods and tools like simulators and e-learning have been adopted, direct hands-on experience with human cadavers remains irreplaceable for medical and surgical education. The absence of clear legislation governing cadaveric dissection has limited availability for teaching and research, resulting in economic burdens for universities and individuals seeking proper surgical training. To address this issue, Law No. 10/2020 and the recent implementing decree were introduced in Italy, providing detailed legislation on the donation of bodies for educational and research purposes. The law emphasizes the importance of respecting the donor’s specific choices and aligns with constitutional principles promoting culture, research, and health protection. However, some critical issues related to consent procedures, duration of body availability, and preservation of anatomical parts remain. Additionally, the law’s dissemination among the population needs improvement. Future optimization could include allowing donors to choose the timing of body donation and considering different timeframes for body availability. Furthermore, the implementation of consent procedures could be simplified to increase donations. The law should also address the need for appropriate reception centers and allocate resources for effective dissemination. Despite these challenges, Law No. 10/2020 represents a significant step forward in enhancing medical-surgical training, scientific research, and the overall quality of patient care in Italy.

## 1. Introduction

The teaching and the scientific research on cadaveric material for medical students, healthcare professionals, and especially specialists and residents in surgical disciplines, still represents an indispensable need rooted in the history of medicine. With the birth of modern medical schools in Italy, the need for and importance of cadaveric dissection in learning anatomy has not taken a back seat. On the contrary, it is an activity that, hand in hand with scientific progress, confirms and strengthens its importance.

Notably, in many anatomical theaters (particularly in Naples and Padua, Italy), stand ancient plaques, that, in Latin, emphasize the importance of post-mortem practice for education, study, and research: “Oh death, I will be your death” (Ero mors tua, o mors), “This is the place where death takes pleasure in assisting life” (Hic est locus ubi mors gaudet succurrere vitae).

The substantial legislative insufficiency has meant that in Italy this practice has been considerably neglected or carried out with significant financial burden, in sharp contrast to what has occurred in other countries.

Dummies as well as virtual three-dimensional or animal models can be a complement but not an alternative to cadavers in surgical training. Current medical training, especially in the surgical field, can rely on innovative methods and tools such as the use of video trainers, simulators, e-learning, and multimedia. While these new technologies have been widely adopted, they cannot be universally applied. The scientific literature, however, suggests that direct hands-on experience with the human body remains important for medical and surgical education [1,2,3,4,5,6,7]. In this context, the new technologies should be considered complementary to hands-on experience [8]. In fact, post-mortem anatomical dissection is not only the means to directly observe the human body: it allows for basic and advanced anatomical and surgical learning, enabling the exploration of new techniques and the refinement of complex ones.

Anatomical dissection on cadavers, of course, requires clear legislation that governs it and establishes its legal boundaries, also framing it from a bioethical perspective. Moreover, in several countries such as the United States, the United Kingdom, France, Germany, and Austria, universities and schools of medicine have their own programs for the donation of bodies, with serious strategies for making the public aware of the importance of cadavers for research and learning in medicine.

In Italy, until 2020, there was no complete and detailed legislative discipline in this regard. The use of cadavers or their parts and tissues for training and scientific research was regulated by rules that were outdated and without concrete operational indications, such as the Decree of the President of the Republic no. 285 of 10 September 1990 and, before that, the Royal Decree n.1592 of 31 August 1933 (in practice, the destination of unidentified and/or unclaimed bodies for scientific purposes was authorized) [1,2]. This significantly limited the availability of bodies or their parts and tissues for teaching, training, and research. This possibility was restricted to rare programs promoted by Italian universities, subject to specific and exceptional authorization.

The lack of body availability, supported by legislative gaps, has resulted not only in repercussions on educational and research needs but also in significant economic consequences for universities, individual medical practitioners, and students. On one hand, academic institutions have been compelled to allocate significant financial resources, amounting to thousands of euros, to import anatomical specimens from foreign countries, solely to facilitate anatomical dissection for educational and research purposes. On the other hand, aspiring students and physicians seeking proper training in surgical or anatomical practices have been burdened with the personal financial responsibility of traveling to countries where cadaver dissection has long been regulated for educational purposes and integrated into educational curricula [1,2].

## 2. Filling the Gaps: Italian Law n. 10/2020 and the Presidential Decree of 10 February 2023

The Law of 10 February 2020, No. 10 [3], and the recent implementing decree (Presidential Decree of 10 February 2023) [9], were issued precisely to fill this legislative gap in Italy, in line with the need to enhance the quality of medical-surgical training and increase research and scientific progress, thereby improving the quality of knowledge and care that future patients will receive. It is an organic and detailed piece of legislation on the donation of bodies, or their parts and tissues, which ensures that the specific choices of the donor are fully respected and appropriately valued.

Law no. 10/2020 consists of ten articles structured as schematized in the Table 1.

The implementing decree provides operational guidelines regarding what is stipulated by the law, with particular reference to the exclusion criteria, methods and timelines for preservation, transportation, utilization, and return of bodies, as well as the dissemination of the law.

As stated by the Italian National Committee for Bioethics in 2013 [10], this law finds “an indirect foundation in the constitutional principle of promoting the development of culture and research (Article 9), especially when this is functional to the protection of health as a fundamental right of the individual and an interest of the community (Article 32)”. Moreover, as for the donation of organs and tissues for transplantation purposes, it clearly aligns with the principle of social solidarity, as set forth in Articles 2 and 3 of the Italian Constitution, and the related “duty of accountability” of the community.

Law no. 10 of February the 10th, 2020 provides the possibility of post-mortem donation of one’s body or body parts for educational, study, and research purposes through the expression of an advance directive, similar to that provided for by the law that, in Italy, regulates advance treatment directives in the clinical setting (Law No. 219/2017) [11]. This means that the donor is required to declare consent through a public deed, an authenticated private document, or a private document delivered to the civil registry office. The declaration containing the consent is then transmitted to the same database that receives advance treatment directives (Decree of the Ministry of Health 10 December 2019, no. 168) [12]. Law No. 219 of December the 22nd of 2017 allows for the possibility of appointing a representative (referred to as a “trustee”) to act on behalf of the settlor in relations with physicians and healthcare facilities. On the other hand, Law No. 10/2020 establishes the appointment of this figure as mandatory. Specifically, the trustee is responsible for informing the physician who ascertains the death about the existence of consent—expressed during life—to the donation of one’s body or body parts to science.

The recent implementing decree (with no small delay compared to the three-month timeframe originally envisaged) identifies the reasons for excluding body donation to science and the cases in which reference centers can refuse to accept them. Furthermore, it regulates the operational steps that must be followed from the moment of death until the body arrives at the reference center, as well as the terms for returning the body to the family for cremation and/or burial. In addition, while the regulation provides for initiatives to inform healthcare personnel and citizens about the provisions of Law No. 10/2020, it does not refer to the promotion of specific financial allocations.

Law No. 10 of February the 10th of 2020 has generally been well-received in the Italian scientific community; however, several critical issues related to operational guidelines, the dissemination of the law, and the methods of expressing consent have been highlighted by the scientific community [13]. Even now, despite the promulgation of the implementing decree, some ethical, operational, and economical issues remain.

The law, as currently formulated, seems to prioritize attention toward a small group of end-users, such as surgeons. Some of the exclusion criteria and the limited duration of the availability period for bodies have been considered inadequate for the purposes of study, education, and scientific research that inspired the law. The time limit within which the body must be returned to the family is set at only twelve months. While this chronological indication satisfies the need to return the body to the family for a dignified burial, allowing for the organization of training trials involving operational practice, it is quite restrictive for didactic and research purposes. This is especially true considering the technical time required for the reception of the body by the facility.

In order to extend the duration of body availability for scientific purposes, it could be possible to allow the donor to decide the time of post-mortem donation of their body or body parts. This provision could represent a future opportunity for legislative optimization.

## 3. Discussion

The object of the present report was to trace the evolution of an Italian roadmap about the present juridical regulations for the scientific use of cadavers. A recent survey of Latin American Anatomists’ point of view stated that, in the surveyed countries, dissection results were most valued as an “Instrument for professional training”, “Instrument to develop professional skills”, and “Source of medical research” [14]. As has been stated in a previous paper by Italian researchers, “In correspondence with the historical religious evolution, another important and determinant factor on the limitations for the use of cadavers is that tied to endemic prevention that characterized the public health during the nineteenth and twentieth centuries; protection of the dead entailed the protection of the public health more than pity and respect. Hence, the religious and historical mentality have deeply influenced the current Italian laws” [15]. Therefore, when the possibility of donating one’s own body, or part of it, is discussed, it must be remembered that this is not just an opportunity for teaching and medical training. The effective functioning of health systems is based on these assumptions too. From this point of view, the donation of one’s body to science is therefore also a public health issue.

Regarding the operational side of the Law. No10/2020 and the presidential decree of 10 February 2023, unless there are specific ethical or religious requirements (for example, in Zulu culture, a series of rituals are performed on the deceased body that end after one year from the death, significantly affecting participation in body donation programs) [16,17], it may be indeed appropriate to follow the examples of states where body donation programs have different and longer timeframes.

In France, there are no time limits at all (Code général des collectivités territoriales, 1996), in the United Kingdom, the limit is set at three years (Art. 5 of Human Tissue Act, 2004), in Turkey, at five to seven years (and is further extended if the bodies are displayed in museums or collections), and in Belgium, the limit is set at one year but can be extended in case of need [18,19,20,21].

It could also be considered that for students of courses such as medicine and surgery, whose duration in Italy is set at 6 years, the fixed limit of 1 year could be inappropriate for the purposes of continuous education. Furthermore, cadaver training sessions, especially in the field of surgery, often involve the participation of internationally renowned specialists. Given the current scarcity of available bodies, the busy schedules of these international experts, and the technical–organizational requirements, the predetermined time limit of 1 year might also prove inadequate in such cases.

It has been stated that “in body donation, the whole body is used, and the corpses are not returned to family and friends. When the body is no longer of use for the anatomical institutes, they are disposed of by cremation”. A Dutch survey conducted by Bolt et al. on organ donors may shed some light on this matter, revealing that family influence plays a role in individuals’ decisions to donate their bodies, and interestingly, this influence also appears to work in the opposite direction [22].

Regarding ethical values, it is of fundamental importance to list the most significant arguments for the debate and the balancing values. So, informed consent affirms that the bodies routinely used in teaching and research will be those donated specifically for these purposes. The use of unclaimed bodies will be viewed as ethically compromised [23]. Body donation, motivated by the need to be useful after death, can be considered an act of contemporary solidarity. From a sociological point of view, it can be explained in a change in solidarity attitudes; individuals no longer need solidarity to survive, but it has become a conscious choice [22]. We can assume that donors’ motivation is more likely to stem from a combination of a desire to help and a sense of personal reward rather than pure altruism [22]. In this regard, it is therefore no coincidence that different campaigns, both for organ or whole-body donations, strongly rely on the spiritual or altruistic aspect of the donation itself, using mots or slogans that imply that the donor is, or can become, a good and admirable person by making his body a gift to society (e.g., the “be an angel” campaign) [24].

Again, it is necessary to reflect on the need for the whole sphere of activity to move in a non-commercial context. A delicate area, where a further slippery slope comes into play, is that of the relationship with family members, made up of dynamics laden with affection and fears for the body, as well as uncertainties related to the timeframes in which these activities are carried out. Added to this is the cultural problem, which is first and foremost an ethical issue to be placed in the social context. Anatomists must convey to all staff and students an awareness of working in the atmosphere of the priceless gift of donations, seeing dissection as an ethical activity in which everyone is a responsible participant [23]. 

The Italian Law of 10 February 2020, no. 10, and Presidential Decree no. 47/2023 state that the donated body must be kept in the morgue for the 24 h following the determination of the death, even if performed with electrocardiographic criteria (ECG recording continues for 20 min) or according to the detailed modalities of the determination of brain death (in subjects undergoing resuscitation care).

However, as is well known in forensics, postmortem brain degradation begins a few minutes after death [25]. The legal obligation to keep donated bodies in the morgue for 24 h, therefore, does not guarantee the optimal preservation of some anatomical parts and tissues studied in the neuroanatomical and neuropathological field [26,27]. This may not allow the full use of bodies and tissues for training and scientific research purposes.

Regarding the exclusion criteria laid down in the implementing decree, we agree on the prohibition of the donation of bodies entrusted to the judicial authority and suicides, as this responds to the possible need to submit them to post-mortem examinations. Equally, it is allowable that the prohibition also extends to those who have died abroad, as there are understandable organizational problems and economic burdens in the international transport of the deceased. Moreover, in these circumstances, the time required by bureaucracy would prevent the availability of donated bodies in an acceptable time. 

According to the implementing decree, the bodies of subjects affected by HIV, HBV, HCV, tuberculosis, syphilis, transmissible spongiform encephalopathies, assistance-related infections—in cases where infection is the sole or prevalent cause of death—antimicrobial resistant infections, SARS-CoV-2 infection—including likely cases, suspected, and confirmed—emerging infections, and bodies undergoing recent treatments with radionuclides or affected with particular pathologies that can expose health professionals to serious risk, are excluded for prudential purposes.

Indeed, there is no doubt that some pathogens, especially if of unknown origin, may pose a risk to the health and life of health professionals involved in autopsies [28]; in this sense, autopsies can allow a targeted and thorough study of these pathogens, however, the use of bodies for scientific purposes does not justify exposure to any microbiological risks, especially—but not only—with regard to cases of SARS-CoV-2 infections [29,30].

If it is reasonable that the bodies of subjects who died in violent circumstances should be stored for the needs of future possible investigations, it is unclear why subjects that died from natural causes dissected for epidemiological and statistical purposes cannot be used for study, training, or research. The same applies to bodies with severe mutilation and extensive open wounds of a post-traumatic nature.

Various reflections can also be made about the issue of consent to the donation of one’s own body or of parts of it and tissues.

As mentioned above, Law No. 10/2020, states that consent to the donation of one’s body for scientific purposes is given in the manner as provided in Law No. 219/2017. Similarly to the rules of Law No. 91/1999 on the donation of organs and tissues, it is therefore not foreseen that legally incompetent people would have the possibility of donating their bodies for scientific purposes. For minors, consent to the post-mortem donation of the body or tissues must be expressed by both parents with parental responsibility, or guardians, or designated persons [31].

In addition, it is extremely important to stress the fact that the donation of one’s own body or parts thereof for training, study, and research purposes is compatible with organ and tissue donation. Thus, it is possible to fully exploit the altruistic gesture of those who decide to donate both their organs or tissues pursuant to Law No. 91/1999, and both their own body or parts of it and tissues pursuant to Law No. 10/2020. 

The way in which informed consent is collected under the law is also particularly complicated and risks reducing the number of donations due to bureaucratic issues and/or lack of information. To promote donation and, consequently, to increase the availability of bodies for scientific purposes, one could adopt—instead of a public act—an authenticated private writing or a private writing delivered to the registry office as required by L. 219/2017, the same method that is already used in Italy and many other countries to express consent to organ donation after death in order to provide consent to the postmortem donation of the body for scientific purposes. Therefore, it would be sufficient to simply sign a specific form when issuing or renewing the identification document [32].

In the United States, there is a lack of specific laws or official guidelines on what information needs to be included in the donation form related to the donation of a human body. However, to address this issue, the American Association of Clinical Anatomists (AACA) developed the “Best Practices Guide for Donation Programs” (AACA, 2017). These guidelines serve as a comprehensive framework, delineating the recommended and optimal practices to be adopted by donation programs to ensure transparency, ethical conduct, and standardization in the donation process. Zealley et al. underline how “greater collaboration among anatomical societies and their members would serve to improve the level informed consent provided to donors and their families. Besides the items recommended by the AACA, more can be done to ensure a higher level of informed consent. However, it is best to start with what is currently recommended” [33].

According to the law, donors do not have the possibility to choose which center to rely on and for how long to donate their body or parts of it after death. These shortcomings are in contradiction with the right to self-determination of the subject, promoted by medicine and recent jurisprudence. According to the implementing decree, the doctor who ascertains the death, with the news of the will of the donor acquired by the fiduciary, identifies the competent reference center and initiates the procedure for the body. Only at a later stage does the reference center verify the presence of informed consent in the dedicated database [12]. Therefore, the presence of the fiduciary at the time of death of the disposer is assumed so that the wishes expressed in life by the subject are respected.

Moreover, in the case of the advance directives, the law provides that there may be a discrepancy between the wishes expressed in life by the subject and the factual situation that occurred (for example, very old directives or scientific progress not foreseeable at the time of the drafting of the directives).

The intermediation of the fiduciary may be essential in such situations. In the case of the donation of the body to science, this possibility is not contemplated. 

The valid consent expressed by the person when he was alive cannot be subject to interpretation by either the doctor or the fiduciary. This consent, as required by the rule, can also be revoked at any time. 

Further criticality about the operation of the law concerns the reference centers for the reception of bodies. These centers must meet the requirements laid down by the Italian Ministry of Health. These requirements include compliance with health and safety standards at work, the availability of areas dedicated to the stationing of bodies, coffins, and equipment, the presence of spaces for training, and the availability of specific procedures for the management of the body, as well as accessibility requirements. To date, there are only seven authorized centers in Italy, of which five are in the north, one is in the south, and one is in Sardinia. This could have important operational and management implications regarding the transport and storage of bodies [34].

Therefore, it is essential that university hospital facilities become suitable for the reception of bodies as soon as possible. The current situation may be likely to favor the paradox of having bodies available for scientific purposes, but no suitable structures to receive them.

The insufficient diffusion of the law inevitably leads to poor participation in donation programs of the body for scientific purposes. The lack of effective dissemination of Law No. 10/2020, aside from the well-known COVID-19 pandemic’s impact, can be due to several factors, including a lack of targeted information campaigns, poor communication by competent authorities, and limited public awareness. This results in many individuals not being aware of the importance, and even of the possibility, of donating their bodies or parts of it for training, study, and research purposes.

Italian anatomists, in fact, in 2021, pointed out in a position paper how “critical issues arise concerning the learners, the type of training and teaching activities that can be planned, the position of the academic anatomy institutes, the role of family members in the donation process, the duration of the donation, the eligibility of partial donation, or the simultaneous donation of organs and tissues to patients awaiting transplantation” [13].

Unfortunately, in the last three years, also due to the COVID-19 pandemic, the dissemination of the Law has not been as effective as could be hoped. To date, this is perhaps the most critical issue. It would therefore be appropriate that of the EUR 4 million allocated for the years 2021, 2022, and 2023 with Law No.178 of 2020, a part is dedicated to the dissemination and promotion of the law among the population [35]. At present, the Italian Minister of Health has not yet defined the criteria and methods for the allocation of these resources.

Conclusively, we believe that this law and its implementing decree have been important for educational advancement, remembering, however, that a combination of teaching tools, both dissection and the use of computer resources, has been repeatedly referred to as superior for learning purposes compared to dissection alone [36]. Similarly, Bergman et al., in a review on the subject, reiterate the same conclusions, “...we would recommend that future research should focus on what and how students learn from dissection and other teaching tools before drawing conclusions in favor of the either method” [8]. This should lead us to also value complementary methods and new techniques that can well complement and integrate cadaveric dissection [37,38,39,40]. To be noted is how “some “modern” anatomists have welcomed the arrival of these novel methods while other, more “traditional,” anatomists have fought to maintain the use of cadaveric dissection” [41].

## 4. Conclusions

Law No. 10/2020 and the implementing decree referred to in D.P.R. 47/2023 are certainly an important novelty in the Italian legislative landscape that regulates a yet well-established practice in other European and non-European countries. This can be considered a turning point both in terms of the quality of anatomy teaching and training, especially of surgeons, and in terms of scientific progress, as well as considerable economic savings for public health facilities, universities, and individual professionals [15].

As has been stated, “anatomical knowledge is too important to future doctors to leave questions about the best way of teaching it to emotional discussions or to the educational fashion of the day” [42].

With this study, we wanted to highlight some critical ethical, operational, and economic issues that emerged from the analysis of Law n. 10/2020 and the implementing decree. For the future, it is desirable that these critical points will be considered in Italian case law. In this manner, the twofold objective of best honoring the memory and the donor’s wishes during their lifetime, and above all, advancing scientific progress, could be pursued. In order to make the donation of the body for study, training, and research purposes an established practice, so as to promote scientific progress and the quality of care, priority should be given to initiatives to disseminate the content of the norms examined [43].

## Figures and Tables

**Table 1:** Contents of the law 10/2020.

Article	Content
Article 1	Matter of the law.
Article 2	Promotion of information.
Article 3	Expression of consent.
Article 4 and Article 5	List and activities of reference centers for the preservation and use of the bodies.
Article 6	Timelines and methods for returning the body to the family.
Article 7	Regulation of monetary donations for the purposes promoted by the law.
Article 8	Adoption of implementing regulation.
Article 9	Financial provisions.
Article 10	Repeal of previous regulations.

## Data Availability

No new data were created or analyzed in this study. Data sharing is not applicable to this article.
